# Bibliometric Analysis of Randomized Controlled Trials on Yoga Interventions for Cancer Patients: A Decade in Review

**DOI:** 10.7759/cureus.58993

**Published:** 2024-04-25

**Authors:** Selvaraj Giridharan, Nagaraj V Kumar

**Affiliations:** 1 Oncology Department, Tawam Hospital, Al Ain, ARE; 2 Emergency Department, Tawam Hospital, Al Ain, ARE

**Keywords:** review, bibliometrics, randomized controlled trials, complementary therapies, cancer, yoga

## Abstract

This literature review presents a bibliometric analysis of the randomized controlled trials conducted between 2014 and 2023 on the potential benefits of yoga as a complementary therapy for cancer patients. To conduct this analysis, we searched medical and scientific databases, such as Scopus, Cochrane, and PubMed, using relevant keywords. Our search yielded 58 clinical trials involving 4,762 patients, which indicates a growing trend in this field of research. The studies we reviewed mainly focused on breast cancer patients and demonstrated the adaptability and versatility of yoga, offering a ray of hope and optimism. Among the various styles of yoga, Hatha yoga was the most frequently practiced style in these clinical trials. The analysis we conducted reveals that yoga interventions have a promising role in cancer care and can be a valuable complementary therapy for cancer patients. However, significant gaps and limitations still need to be addressed in this area of research. For instance, more rigorous and diverse investigations are needed to further establish the potential benefits of yoga interventions for cancer patients. Additionally, the standardization of yoga interventions is crucial to optimize therapeutic benefits. By addressing these gaps and limitations, we can further enhance the potential of yoga as a complementary therapy for cancer patients.

## Introduction and background

Cancer significantly burdens global health, with projections indicating a persistent increase in prevalence over the next two decades [1,2]. In 2019, 23.6 million new cancer cases and 10.0 million cancer-related deaths were recorded globally. Excluding non-melanoma skin cancer, the numbers stand at an estimated 17.2 million new cases and 9.97 million deaths. From 2010 to 2019, new cancer cases rose by 26.3%, and total cancer deaths increased by 20.9%, highlighting an upward trend in cancer’s impact on mortality and morbidity. Amid this growing challenge, cancer survivors increasingly turn to complementary and alternative medicine (CAM) to alleviate symptoms, enhance quality of life, and manage stress [3-5]. CAM encompasses a range of interventions, including yoga, that aim to address the holistic well-being of individuals, extending beyond conventional cancer treatment [6]. The widespread use of CAM among cancer survivors emphasizes the need for an integrated care approach that synergizes with traditional medical treatments [7].

Yoga, an ancient Indian practice, has emerged as a prominent complementary therapy in cancer care, recognized for its potential to improve health outcomes and manage treatment-related symptoms [8]. Distinct from religious or belief systems, yoga is a science comprising various techniques such as asanas, pranayama, meditation, and ethical principles. These practices foster a harmonious balance between body, mind, and spirit, contributing to improved physiological and psychological states [9,10]. Yoga’s holistic benefits have been documented across various health conditions, including chronic pain and heart, lung, and neurological diseases, making it especially relevant for cancer patients navigating the complexities of their treatment [11,12].

Recent studies have begun to integrate yoga as a supportive therapy in cancer care, revealing its feasibility and benefits in enhancing physical functioning, reducing fatigue, and improving sleep quality and psychological well-being [13-15]. However, the broad range of yoga interventions and methodologies and the subjective nature of well-being assessments necessitate a systematic approach to synthesize and evaluate the existing literature. Bibliometric analyses are crucial in this regard, providing a quantitative overview of scientific publications to assess research trends, productivity, and impact. These analyses effectively navigate the complex literary landscape, identifying critical articles, influential authors, leading institutions, and contributing countries. Through this thorough examination, bibliometric analyses establish a foundational framework that enables a more profound understanding of yoga’s evolving role in cancer care and also guides future research directions.

## Review

Objective

This bibliometric analysis aims to systematically review randomized controlled trials (RCTs) that have explored yoga as an intervention for cancer patients over the past decade. By employing a bibliometric approach, we seek to map the research landscape, uncover core themes, identify gaps, and evaluate the studies’ impact and methodological quality. Our objectives are multifold: to assess the growth and volume of RCTs on yoga for cancer patients, understand the geographical distribution and collaboration networks, evaluate citation impacts, and pinpoint the main thematic areas of investigation. This analysis will not only shed light on the robustness of the evidence base supporting yoga as a therapeutic intervention but also enrich the discourse on the role of complementary therapies in oncology, providing a comprehensive overview of yoga’s potential as a supportive care option for cancer patients.

Methods

This bibliometric analysis follows the Preferred Reporting Items for Systematic reviews and Meta-Analyses (PRISMA) guidelines and systematically reviews RCTs that focus on yoga interventions for cancer patients over the past decade. The methodology is detailed below.

Data Sources and Search Strategy

We conducted searches in major medical and scientific databases, including PubMed, Cochrane Library, and Scopus, to ensure comprehensive coverage of the literature. A combination of keywords and MeSH terms related to “yoga,” “cancer/oncology,” and “randomized controlled trial” were used. Boolean operators (AND, OR) facilitated the construction of comprehensive search strings; for example, (“yoga” OR “mind-body therapy”) AND (“cancer” OR “oncology”) AND “randomized controlled trial.” Searches were filtered to include studies published in English within the specified 10-year period between January 2014 and December 2013.

Inclusion and Exclusion Criteria

Only RCTs that specifically investigated yoga as an intervention for patients diagnosed with cancer were included. Studies needed to report on measurable clinical outcomes related to physical, psychological, or quality-of-life aspects. Non-RCTs, observational studies, reviews, commentaries, and studies not focusing on cancer patients were excluded. Studies where yoga was not the primary intervention were also excluded.

Data Extraction and Management

Two independent reviewers extracted data using a standardized form, capturing information on publication year, authors, country of origin, sample size, cancer type, type of yoga intervention, duration and frequency of the intervention, outcome measures, and main findings. Any reviewer discrepancies were resolved through discussion. Hatha yoga, the style most frequently mentioned in the manuscript, is traditionally focused on physical postures (asanas), breathing techniques (pranayama), and meditation. Although many studies do not specify an exact style of yoga, they describe using a combination of these three key elements, which aligns with the practices central to Hatha yoga. For our trend calculations and discussion, any mention of a combination of asanas, pranayama, and meditation has been categorized under Hatha yoga. This categorization helps standardize the interventions across studies for clearer analysis and trend identification.

Results

**Figure 1:**
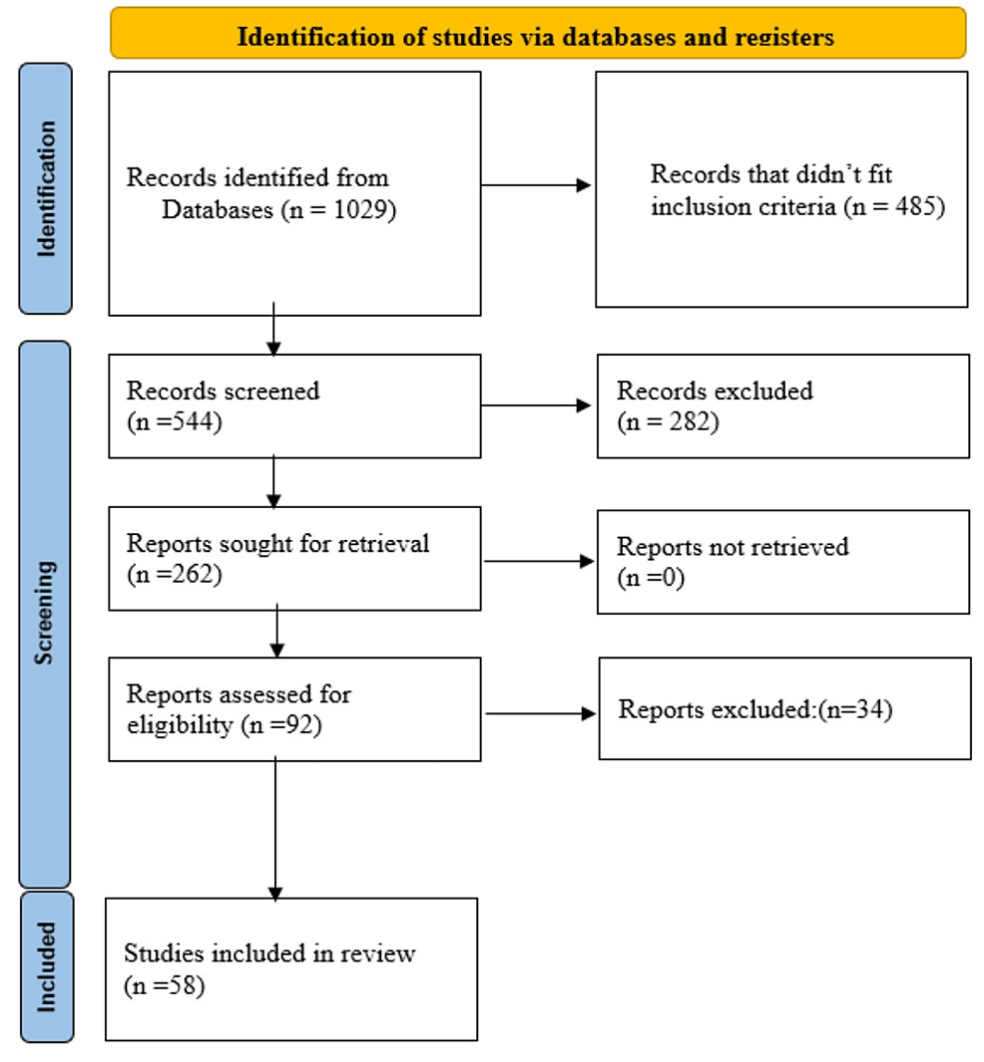
Summarized search strategy (PRISMA diagram) PRISMA, Preferred Reporting Items for Systematic reviews and Meta-Analyses

The PRISMA chart outlines the systematic process of identifying and selecting studies for a comprehensive review (Figure 1).

**Figure 2:**
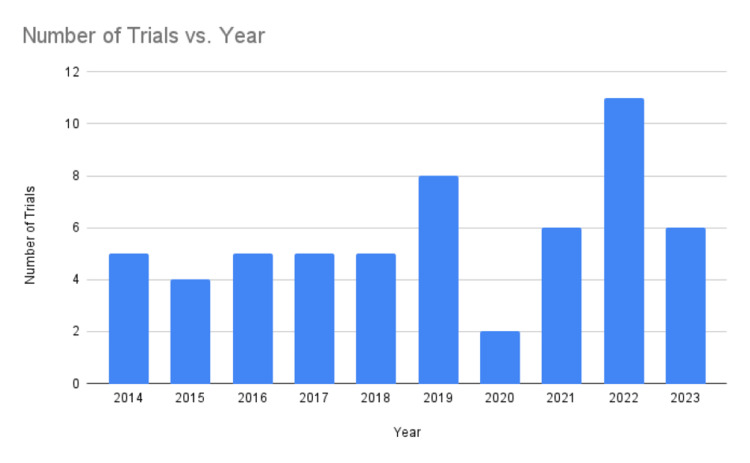
Number of trials per year

In the initial search, we identified 1,029 from the databases. Of these records, 544 were screened, and 282 were excluded as they did not meet the inclusion criteria. This left us with 262 reports that needed retrieval. All 262 reports were appraised successfully, and after further review, 34 reports were excluded. The eligibility of the remaining 92 reports was assessed, and 58 studies were included in the final evaluation [16-73]. The number of clinical trials examining the therapeutic effects of yoga on cancer patients has significantly increased from 2014 to 2023, indicating a growing interest in integrative oncology practices (Figure 2).

In contrast, between 2004 and 2013, only 25 RCTs were conducted during the previous decade, highlighting an impressive 132% increase in the number of clinical trials investigating the role of yoga in cancer care. This remarkable growth reflects a surging interest and recognition within the medical community regarding the potential benefits of yoga as a complementary therapy for cancer patients. In total, 4,762 patients were enrolled in the trials, which highlights the considerable interest in investigating the role of yoga in cancer care. All the trials implemented some form of randomization, either entirely or in comparison to a waitlist control group. Eleven of these trials were pilot studies, demonstrating a systematic and exploratory approach to research design in this emerging field. It is worth noting that only three trials included patients aged 80 and above.

The clinical trials conducted on the role of yoga in cancer treatment covered different stages of the treatment process. A total of 27 trials focused on patients undergoing active chemotherapy, nine on those receiving radiotherapy, and 16 on evaluating the benefits of yoga in the post-treatment phase for survivors. In addition, three trials addressed the needs of caregivers, indicating a holistic approach to patient support networks. The trials covered a broad range of areas, including immune functions and gene expressions (n = 6), psychological factors such as anxiety and depression (n = 24), quality of life (n = 37), treatment-related adverse events (n = 9), sleep quality (n = 8), cancer-related fatigue (n = 8), chemotherapy-induced peripheral neuropathy (n = 3), and cognitive functions (n = 4). These areas of focus highlight yoga’s ability to address both the physical and psychological effects of cancer and its treatments.

**Figure 3:**
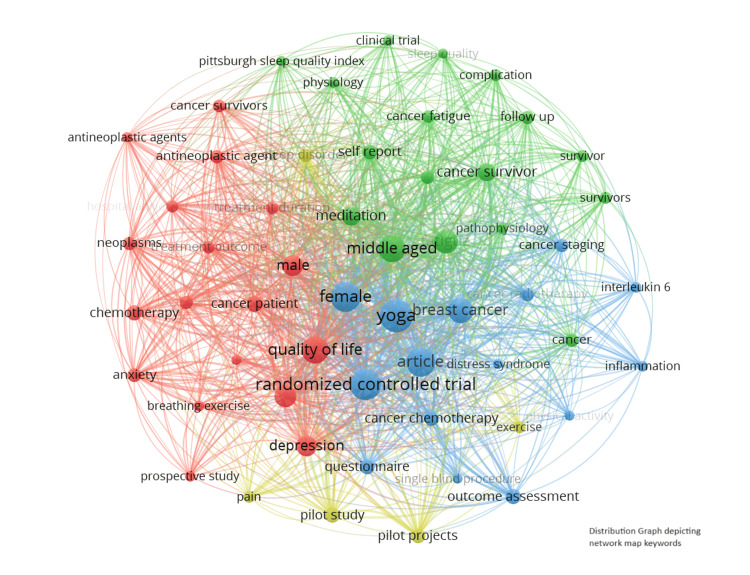
Distribution graph depicting network map keywords

Yoga interventions have been proven to offer numerous health benefits for cancer patients. It has been shown to improve the quality of life, manage treatment-related side effects such as fatigue and nausea, and enhance psychological well-being by reducing stress, anxiety, and depression. Yoga has also been associated with better sleep quality, increased strength and flexibility, and improved pain management. Certain studies suggest that yoga positively affects immune system functioning and helps stabilize specific biomarkers related to cancer progression. The VOS viewer keyword analysis of the included RCTs reveals a strong focus on breast cancer within yoga research, highlighting its integration with standard cancer treatments like chemotherapy and radiotherapy. Studies predominantly investigate the impact on symptoms and quality of life, with a notable emphasis on psychological outcomes such as depression and mindfulness. The research encompasses a diverse demographic, focusing on gender and age-related factors in cancer survivorship (Figure 3).

**Figure 4:**
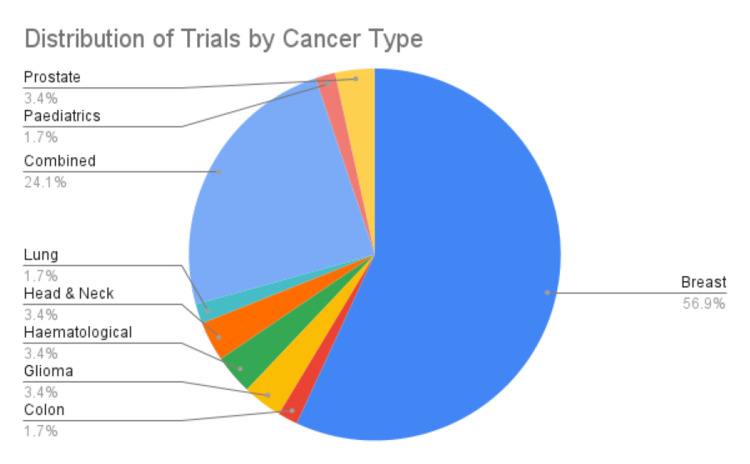
Distribution of trials by cancer type

The number of patients recruited in the trials varied widely in sample size, ranging from as few as 20 participants to as many as 410. This diversity in scale indicates the potential for varying data collection and analysis levels. Breast cancer trials dominate the portfolio [17,18,22-24,27,29,30-32,34,38,39,41,43,45,47,50,51,56,58,59,60-65,68-72], possibly due to the high prevalence of breast cancer and the active survivor communities often engaged in complementary therapies. However, there is a notable disparity in research focus toward cancer types such as colon [26], glioma [52,53], hematological malignancies [25,37], head and neck [16,19], and lung cancer [35], with each category having only one or two studies. The “mixed” category has 14 trials [20,28,33,36,40,44,46,48,49,54,55,57,67,73], which suggests an investigative interest in the general applicability of yoga interventions across different types of cancer rather than site-specific effects. Pediatric [66] and prostate cancer [29,50] are represented with one and two trials, respectively, pointing to emerging, yet still limited, research within these patient populations (Figure 4).

**Figure 5:**
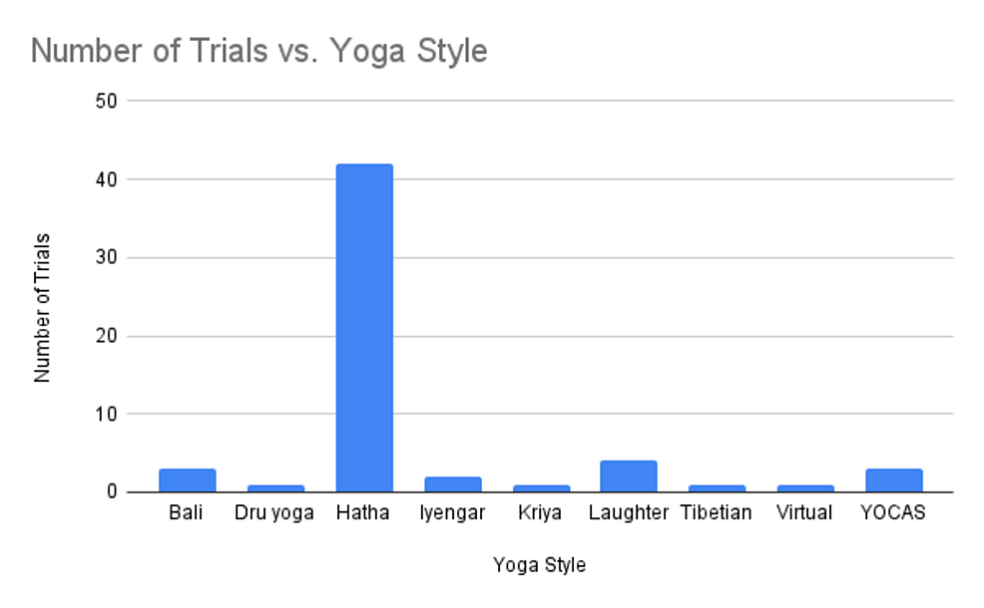
Number of trials vs. yoga style

The distribution of yoga styles in clinical trials highlights yoga’s versatility in the holistic care of cancer patients. Hatha yoga, the most frequently studied style known for its gentle approach, was featured in 42 trials, attesting to its widespread acceptability and adaptability. Notably, the Yoga for Cancer Survivors (YOCAS) program, developed by the University of Rochester Medical Center, was employed in three significant trials enrolling a combined total of 905 patients, highlighting its substantial implementation and potential impact on cancer survivorship. The program caters to the complex needs of cancer survivors by addressing their physical, emotional, and spiritual well-being. Furthermore, the inclusion of less common styles such as laughter, Iyengar, Kriya, Dru, and Tibetan yoga, as well as virtual yoga sessions, points to an evolving curiosity and innovative spirit in the research community, seeking to broaden access to yoga therapy for cancer patients (Figure 5).

**Figure 6:**
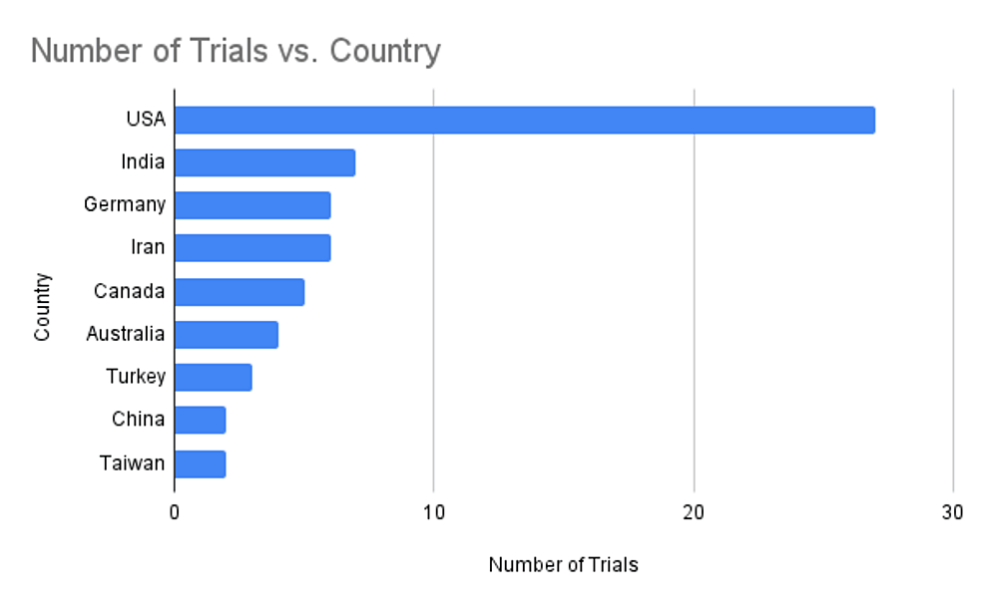
Number of trials vs. country

Research on yoga’s potential benefits for cancer patients is being conducted worldwide, with the United States leading the way. India and other countries have also made significant contributions to this field (Figure 6).

**Figure 7:**
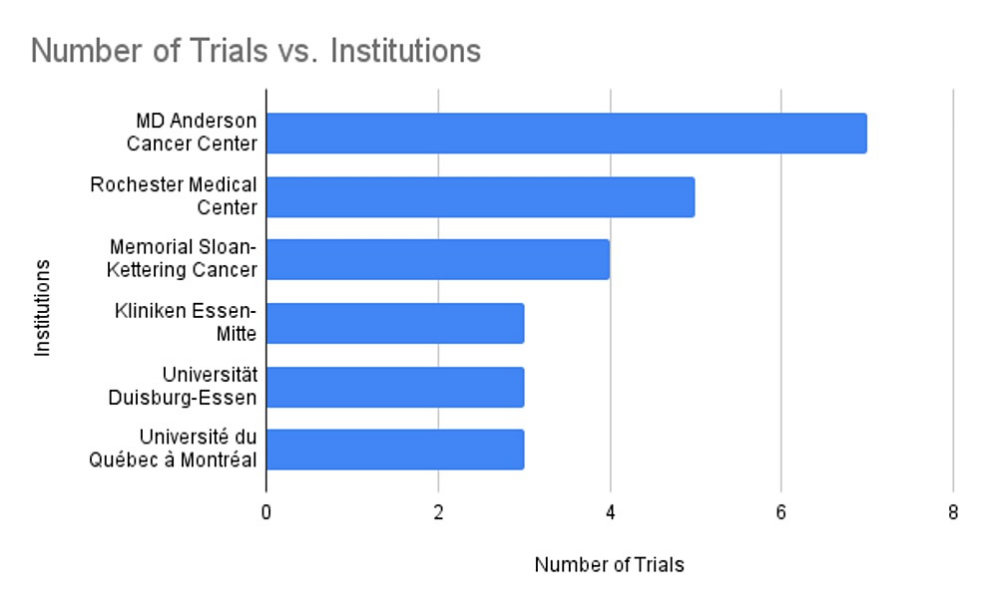
Number of trials vs. institutions

The widespread interest in clinical trials of yoga for cancer patients indicates a global investment in this area. Numerous cancer research centers have been advancing this field, such as the MD Anderson Cancer Center, the University of Rochester Medical Center, and the Memorial Sloan Kettering Cancer Center (Figure 7).

**Figure 8:**
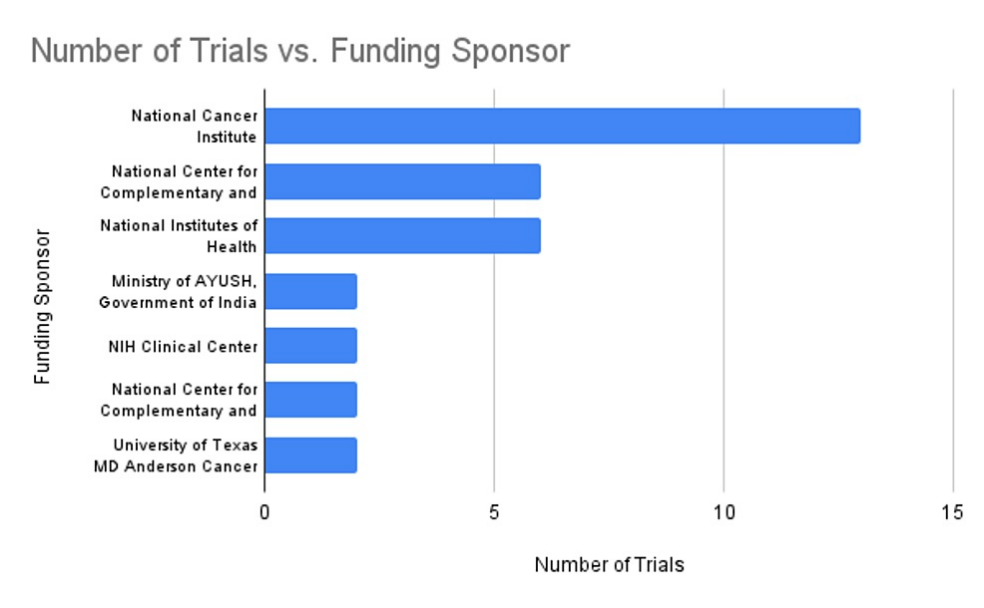
Number of trials vs. funding sponsor

The support of prominent cancer centers and key governmental health agencies in the United States, such as the National Cancer Institute and the National Institutes of Health, reflects a growing recognition of the value of complementary therapies like yoga in a holistic approach to cancer care (Figure 8).

This trend toward embracing supportive therapies alongside traditional treatments affirms the commitment within the scientific community to comprehensive, patient-centered care models that acknowledge the multidimensional needs of cancer patients.

Discussion

Our bibliometric data analysis indicates that there has been a rise in the number of trials examining the effectiveness of yoga interventions for cancer patients in the past decade. This trend reflects a shift toward a holistic care model that considers cancer patients’ physical, psychological, and spiritual well-being. In 2014, the Cramer et al. study provided a comprehensive review of yoga RCTs up until that time [74]. Our analysis builds upon and extends the work of Cramer et al. by focusing specifically on the use of yoga in the context of cancer care. Cramer et al.’s study provided an overview of the impact of yoga on various health outcomes, while our analysis delves deeper into its therapeutic benefits and implementation in oncology. We highlight the increase in research volume over recent years and examine the specific types of cancers studied, the range of yoga interventions used, and the detailed outcomes relevant to cancer patients. Our study also looks at the geographic distribution of research efforts, showcasing how cultural and policy differences influence the study of yoga in cancer care globally. By identifying these nuances, our work contributes to a more focused understanding of how yoga is integrated into cancer treatment protocols and pinpoints areas needing further investigation, thus adding a significant layer of specificity and application to Cramer et al.’s broader analysis.

Our analysis suggests that certain types of cancer, such as breast cancer, and specific outcomes, such as quality of life and fatigue, have received more attention, indicating a focus on areas where yoga’s benefits are most promising. Moreover, the distribution of studies across different geographical areas shows a noticeable concentration of research in Western countries. This trend reflects the influence of cultural acceptance and healthcare policies on the research agenda. However, despite significant progress in the field of cancer research, the studies conducted primarily focus on specific cancer types and age groups, with an underrepresentation of other types and age groups. Standardizing research methodologies and adopting a more inclusive approach are essential to address the gaps and disparities in research. Hence, further research is urgently needed to fill the existing gaps, including diverse studies concerning different cancer types, long-term follow-up studies, and studies from low- and middle-income countries.

The analysis highlights the potential of yoga as a valuable adjunct to conventional cancer therapies. It can improve mental well-being, alleviate symptoms of depression and anxiety, and enhance physical function. Integrating yoga programs into oncology care protocols can enhance patient outcomes and improve standardized quality. Due to the variability in yoga interventions, standardized guidelines are essential for optimizing yoga interventions and achieving the best therapeutic benefits for cancer patients. Consistency in yoga practice allows researchers to compare results, identify the most beneficial practices, and integrate them into guidelines. Clear protocols for yoga interventions, including style, duration, frequency, and postures, must be established. Consistent training and qualifications for instructors are also required. Standardization enables healthcare providers to offer yoga as a therapy with confidence in its ability to support the well-being of cancer patients.

Our review, although comprehensive, has certain limitations. Relying on published RCTs may lead to publication bias, as studies with positive outcomes are more likely to be published. The exclusion of non-English-language studies could have resulted in the omission of valuable research, limiting the analysis’s global perspective. Furthermore, the quality assessment of the included RCTs showed variability in methodological rigor, which could affect the interpretation of yoga as a therapeutic intervention’s efficacy. These limitations, inherent to the methodology, underscore the need for a cautious interpretation of the findings and suggest areas for methodological improvement in future research.

Although increasing evidence supports yoga’s benefits for cancer patients, the current research still has gaps and limitations. Firstly, specific demographics, such as elderly patients and those with less common types of cancer, are underrepresented, which limits the generalizability of the findings. Additionally, most studies are conducted in high-income countries, which raises concerns about how applicable the results are to populations in low- and middle-income countries. Furthermore, the majority of studies are short-term, which means that the long-term effects of yoga on cancer survival and recurrence rates are not well documented. There is also a need for uniformity in the types of yoga interventions studied, which makes it challenging to compare outcomes across trials and establish standardized treatment protocols. Lastly, many studies rely on self-reported measures, which can introduce bias and affect the reliability of outcome data.

## Conclusions

Yoga interventions hold great promise in cancer care, as shown by the increasing number of RCTs, the involvement of prestigious cancer centers, and diverse research areas and yoga styles. However, this field still has significant gaps and limitations, highlighting the need for more rigorous and diverse investigations. Standardizing yoga interventions can help their widespread adoption in oncology, thereby improving the overall care of cancer patients.
